# Challenges and Inconsistencies in Using Lysophosphatidic Acid as a Biomarker for Ovarian Cancer

**DOI:** 10.3390/cancers11040520

**Published:** 2019-04-11

**Authors:** Tsukasa Yagi, Muhammad Shoaib, Cyrus E. Kuschner, Mitsuaki Nishikimi, Lance B. Becker, Annette T. Lee, Junhwan Kim

**Affiliations:** 1 Center for Immunology and Inflammation, Feinstein Institute for Medical Research, 350 Community Dr., Manhasset, NY 11030, USA; tsuyagi-circ@umin.ac.jp (T.Y.); mshoaib1@northwell.edu (M.S.); mnishikimi@northwell.edu (M.N.); lance.becker@northwell.edu (L.B.B.); 2 Donald and Barbara Zucker School of Medicine at Hofstra/Northwell, 500 Hofstra Blvd, Hempstead, NY 11549, USA; ckuschner1@pride.hofstra.edu (C.E.K.); alee@northwell.edu (A.T.L.); 3 Robert S. Boas Center for Genomics & Human Genetics, Feinstein Institute for Medical Research, 350 Community Dr., Manhasset, NY 11030, USA

**Keywords:** lysophospholipids, LC-MS, diagnosis, lipidomics

## Abstract

Increased detection of plasma lysophosphatidic acid (LPA) has been proposed as a potential diagnostic biomarker in ovarian cancer, but inconsistency exists in these reports. It has been shown that LPA can undergo an artificial increase during sample processing and analysis, which has not been accounted for in ovarian cancer research. The aim of this study is to provide a potential explanation about how the artificial increase in LPA may have interfered with previous LPA analysis in ovarian cancer research. Using an established LC-MS method, we measured LPA and other lysophospholipid levels in plasma obtained from three cohorts of patients: non-cancer controls, patients with benign ovarian tumors, and those with ovarian cancer. We did not find the LPA level to be higher in cancer samples. To understand this inconsistency, we observed that LPA content changed more significantly than other lysophospholipids as a function of plasma storage time while frozen. Additionally, only LPA was found to be adversely impacted by incubation time depending on the Ethylenediaminetetraacetic acid (EDTA) concentration used during blood drawing. We also show that the inhibition of autotaxin effectively prevented artificial LPA generation during incubation at room temperature. Our data suggests that the artificial changes in LPA content may contribute to the discrepancies reported in literature. Any future studies planning to measure plasma LPA should carefully design the study protocol to consider these confounding factors.

## 1. Introduction

Ovarian cancer is the fourth leading cause of all cancer deaths despite its relatively low frequency [1]. This high mortality is attributable to the lack of early primary symptoms and reliable biomarkers, which prevent establishing an early diagnosis. Consequently, more than 75% of cases are diagnosed at advanced stages, such as stage III or IV [2,3]. Survival is significantly improved with an early diagnosis: 5-year survival of patients diagnosed with stage I–II versus stage III–IV ovarian cancer is 90% and 30%, respectively [4,5]. Therefore, establishing an early diagnostic biomarker is essential to improving ovarian cancer survival. 

The most commonly studied biomarker for ovarian cancer is cancer antigen 125 (CA-125). However, the utility of CA-125 as a diagnostic marker is limited as it detects approximately 50% of patients with stage I ovarian cancer and cannot distinguish between a benign and a malignant adnexal mass [6,7]. As such, pelvic examination and imaging tests are commonly used to confirm the presence of a pelvic mass. The final diagnosis of a benign or malignant adnexal mass is determined by the pathological examination of a surgically excised tumor [3].

In 1998, Xu et al. first reported the increased plasma level of lysophosphatidic acid (LPA) in ovarian cancer patients compared to patients with benign gynecologic disease, or healthy controls [8]. This promising report prompted numerous follow-up studies testing the diagnostic potential of LPA and other lysophospholipids in ovarian cancer, as well as LPA’s potential role in the development and progression of ovarian cancer [9,10]. Although numerous studies have reported the possibility of LPA as a diagnostic marker, there remains significant discrepancy regarding the utility of LPA in the literature. These irregularities may be caused by the challenges associated with accurately quantifying LPA concentrations.

LPA is a lysophospholipid without a head group. By losing their head groups, any class of lysophospholipid can be converted into LPA [11]. In fact, studies using controlled experiments have shown that LPA can artificially increase during the processing and analysis of blood samples [12,13]. However, the artificial increase of LPA has not been fully elucidated regarding the more complicated procedures of obtaining and processing clinical samples. Clinical research has not yet considered the artificial increase in testing LPA as a potential diagnostic marker. Clearly, there is a gap between the biochemical understanding and the clinical practice of analyzing LPA from patients’ specimens. Without ensuring biomarker stability, numerous studies have affirmed that ovarian cancer resulted in increased plasma LPA levels [8,14,15]. Despite contradictions in these reports, LPA is still considered to be a potential diagnostic marker of ovarian cancer [16,17,18,19,20].

Using an established extraction procedure and LC-MS method, we measured LPA and other lysophospholipid levels in plasma samples obtained from patients with ovarian cancer, patients with benign ovarian tumors, and non-cancer controls. After finding that LPA levels were higher in the control than in the malignant ovarian cancer patient samples, we investigated several factors which could explain this discrepancy, including LPA alteration during incubation, storage, and the concentration of ethylenediaminetetraacetic acid (EDTA). We found that the content of LPA significantly increased during storage when plasma samples were frozen, and that the LPA increase during incubation of blood samples at room temperature was impacted by the EDTA concentration during blood collection. We discuss how these artefactual changes may have interfered with LPA analysis, contributing to the discrepancy of LPA observed in ovarian cancer patients throughout the literature.

## 2. Results

### 2.1. LC-MS Analysis of Lysophospholipids

**Figure 1:**
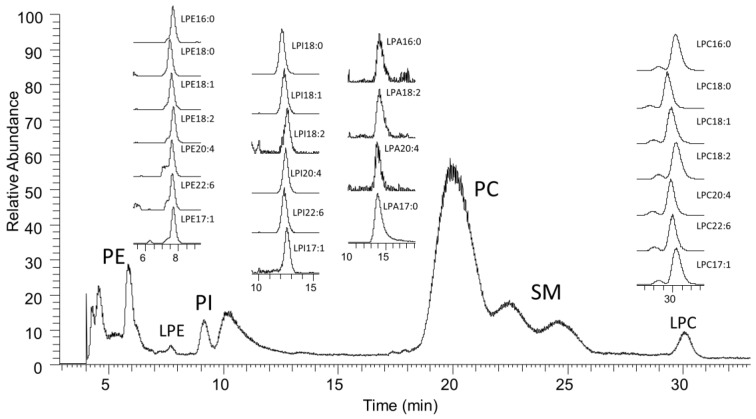
Ion chromatogram of HPLC-MS analysis. The extracted ion chromatograms of individual species of lysophosphatidylethanolamine (LPE), lysophosphatidylinositol (LPI), lysophosphatidic acid (LPA), and lysophosphatidylcholine (LPC) were generated using the corresponding molecular weights at the given retention times compared to standard lysophospholipids.

A representative total ion chromatogram of phospholipid and lysophospholipid in control samples is shown in Figure 1. Extracted ion chromatograms of individual lysophospholipids are also shown in the inserts. Peaks were identified by their retention time, MS, and MS/MS data when compared to standard phospholipids. The total ion chromatogram shows the four most abundant phospholipids present in human plasma, phosphatidylethanolamine (PE), phosphatidylinositol (PI), phosphatidylcholine (PC), and sphingomyelin (SM). Abundant lysophospholipids, lysophosphatidylcholine (LPC) and lysophosphatidylethanolamine (LPE), are also visible in the total ion chromatogram. Less abundant LPA and lysophosphatidylinositol (LPI) were detected in extracted chromatograms. The extracted peaks corresponding to the major lysophospholipid species are shown in the insert of Figure 1. Although clearly shown in this specific sample, which is approximately three years old, LPA(20:4) was barely detectable in most of the samples and LPA(16:0) was not quantifiable in most of the cancer and benign samples.

The retention times of individual lysophospholipids slightly differ depending on the attached acyl chain. Species with a shorter chain length and more double bonds elute slower due to their stronger interaction with the hydrophilic stationary phase. The retention times of phospholipids and lysophospholipids are consistent with previous reports [21]. This class separation removes the chance for artificial conversion of other lysophospholipids to LPA during mass analysis, as previously reported [22]. We noticed that, although there was an overlap between LPI and LPA, we did not detect any peaks corresponding to LPA(17:1), which can be theoretically generated from LPI(17:1). This result confirms that the conversion of LPI to LPA is minimal. Overall, our HPLC-MS method clearly identifies each class of lysophospholipids.

### 2.2. Comparing Lysophospholipids in Control and Ovarian Cancer Plasma Samples

We compared lysophospholipid levels between three separate groups: control, cancer, and benign (Table 1). All of the lysophospholipids measured in our study displayed a linear response in the concentration range. The limit of detection of LPA17:0 is 2.8 pmol and the limit of quantitation is 5.6 pmol. LPA(18:2) was 2.6-fold higher in the control than in the cancer or benign groups. There was no difference between the cancer and benign groups. Although LPA(16:0) was not measurable in the cancer or benign groups, the data clearly shows that LPA(16:0) was also higher in the control than in cancer or benign groups. The control group LPI(18:0) was 3.3-fold higher than in the cancer group, while LPI(20:4) was higher in the cancer than in the control group. The contents of LPC(16:0) and LPC(18:0) were mildly increased in the control compared to the cancer group, whereas LPC containing polyunsaturated fatty acids, LPC(20:4) and LPC(22:6), were higher in the cancer than the control group. The contents of these LPC species in the benign samples were between both groups. In addition, there was no significant difference in the contents of LPC(18:2) between the three groups. There was no difference in LPE(16:0) between the three groups. The LPE(18:0) level was significantly higher in the control than in the benign group, and the LPE(18:2) level was significantly higher in the control than in the cancer group. However, LPE(22:6) was significantly higher in the benign and cancer groups than in the control group, while LPE(20:4) showed an increase in the benign group when compared to the control group.

Overall, both LPA(18:2) and LPA(16:0) were significantly higher in the control than in the other two groups. In the other classes of lysophospholipids, the following trend was observed: species with saturated fatty acids were higher in the control group, while the contents of species with unsaturated fatty acids were higher in the cancer group. These data suggest that, unlike previous reported studies, LPA species that were quantifiable were at higher levels in the healthy control patients when compared with the benign or ovarian cancer patients.

### 2.3. Phospholipids and Lysophospholipids Change over Time

**Figure 2:**
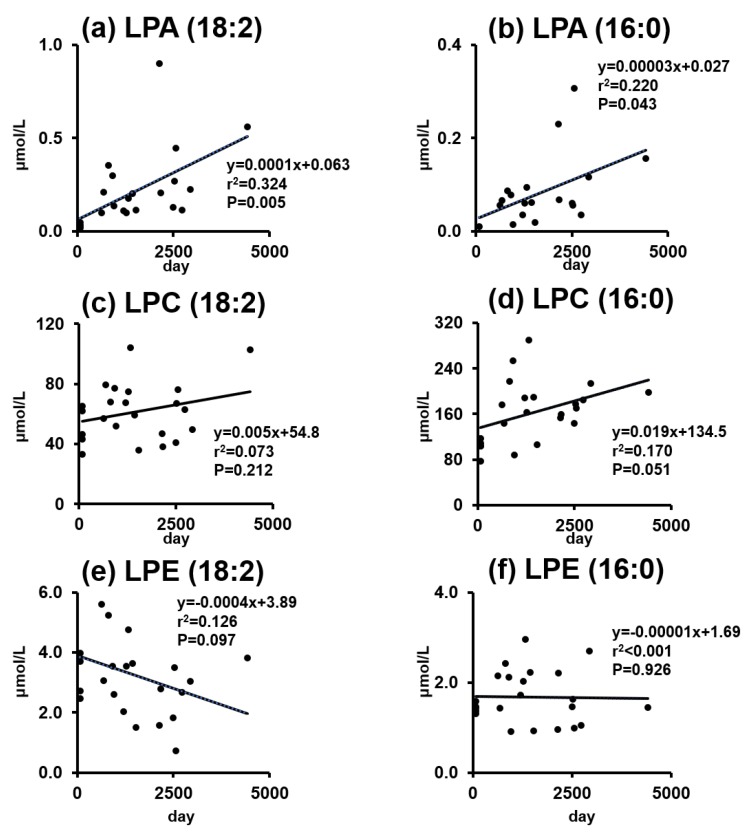
Regression analysis of the individual lysophospholipid species as a function of the duration of storage time in the control samples. The contents of LPA(18:2) and LPA(16:0) were significantly increased as a function of storage times (**a**,**b**), whereas the contents of LPC(18:2), LPC(16:0), LPE(18:2), and LPE(16:0) were not significantly changed (**c**–**f**).

We performed a regression analysis to follow changes in lysophospholipid levels as a function of storage time, using the control samples alongside fresh plasma obtained from healthy volunteers (Figure 2). Applying a linear regression, we found that the contents of LPA(18:2) and LPA(16:0) significantly increased with longer storage times. Although not statistically significant, the changes in LPC(16:0) (*p* = 0.051) and LPE(18:2) (*p* = 0.097) were also noted. The regression of other lysophospholipid species is shown in the supplementary data (Figure S1). Despite some exceptions based on individual lysophospholipid species, the data showed LPI to be the most susceptible to increase with prolonged storage time, closely followed by LPA, while LPE displayed the slowest rate of change with prolonged storage. Collectively, Figure 2 shows that lysophospholipid concentrations changed over time, even when stored at −80 °C, and that LPA content significantly increased as the storage time was prolonged.

### 2.4. Lysophospholipid Changes Depending on Incubation Time, EDTA Content, and Autotaxin Inhibitor

We determined the influence of blood incubation time at room temperature on the changes in LPA content. The typical procedure for treating obtained blood samples requires separation of the blood within 4 h of drawing it and immediately freezing the separated plasma. Therefore, we incubated whole blood for up to 4 h in the presence of 5 mM or 10 mM of EDTA. As a comparison, we also incubated the separated plasma using the same conditions.

**Figure 3:**
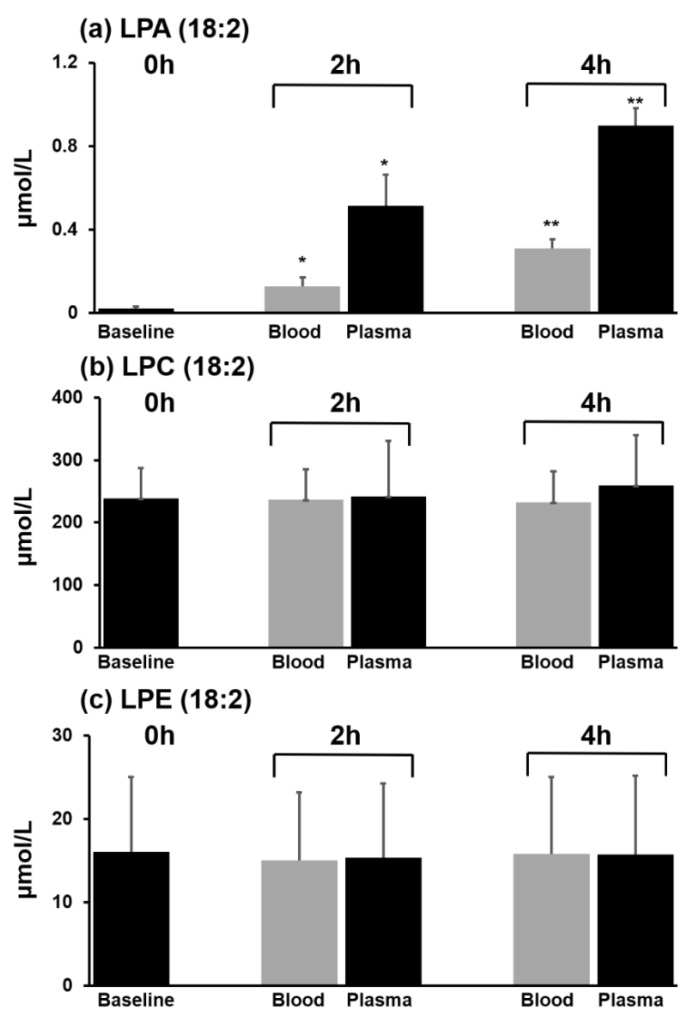
Changes in the contents of LPA(18:2), LPC(18:2), and LPE(18:2) in whole blood and plasma incubated at room temperature with 5 mM EDTA. * *p* < 0.01 for comparison of LPA(18:2) vs. 0 h and ** *p* < 0.01 vs. 2 h. The content of LPA(18:2) was significantly increased both in whole blood and plasma depending on incubation time (**a**), whereas the contents of LPC(18:2) and LPE(18:2) were not changed (**b**,**c**).

Figure 3 shows the changes in LPA(18:2), LPC(18:2), and LPE(18:2) content in the presence of 5 mM EDTA. The content of LPA(18:2) increased to 0.13 µmol/L from 0.02 µmol/L after 2 h, and to 0.31 µmol/L after 4 h of incubation in whole blood. In plasma, we also found that the content of LPA(18:2) increased as a function of incubation time, but to a much greater extent. After 4 h, the content of LPA(18:2) increased to 0.90 µmol/L, which was ~3 times higher than the content found in whole blood incubation (Figure 3a). However, there was no change in the content of the other classes of lysophospholipids. We show the data of LPC(18:2) and LPE(18:2) as an example (Figure 3b,c). The increase in LPA content was smaller in the presence of 10 mM EDTA, which was approximately 30% lower than the increase found in 5 mM EDTA (Figure S2). Together, these data indicate that EDTA interfered with the artificial generation of LPA during incubation at room temperature and that the increase was dependent on the concentration of EDTA.

**Figure 4:**
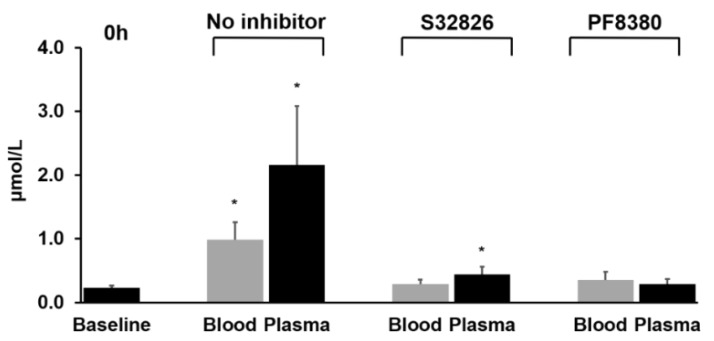
Changes in the contents of LPA(18:2) in whole blood and plasma after the addition of each autotaxin inhibitor and incubation at room temperature for 4 h. * *p* < 0.01 for comparison of LPA(18:2) vs. baseline.

To investigate the role of autotaxin (ATX) in the increase in LPA content, we added 1 µM of an ATX inhibitor, either PF-8380, or S32826, to blood samples containing 5 mM EDTA and incubated the samples at room temperature for 4 h (Figure 4). PF-8380 maintained the LPA levels close to the baseline during the 4 h incubation. In spite of the slightly elevated LPA level compared to the baseline with S32826 in plasma, both inhibitors almost completely inhibited the artificial increase of LPA in whole blood and plasma to acceptable levels. Overall, out of all of the lysophospholipid species analyzed, only the content of LPA increased with incubation time. This increase was greater with lower concentrations of EDTA and was significantly prevented by the addition of ATX inhibitors.

## 3. Discussion

We found that the plasma content of LPA was significantly higher in the control than in the ovarian cancer groups using stored plasma samples. This finding adds more contradiction to existing reports on LPA’s relation to ovarian cancer, benign ovarian masses, and controls. The results from previous studies on LPA in ovarian cancer are summarized in Table S1. A group of studies reported significantly greater LPA levels in ovarian cancer over control plasma, with an average LPA content of 4–9.3-fold greater in ovarian cancer samples and 2–3-fold greater in benign ovarian masses when compared to control plasma samples [8,23,24,25,26,27]. However, another group of studies reported only a mild increase of 1.7–2.9-fold in LPA content in ovarian cancer when compared to control samples [14,15,28,29,30,31]. Moreover, the relative content of LPA in the benign group was contradictory in these groups, with some reporting no differences in LPA content between benign masses and control patients [15], while others found no difference between benign adnexal masses and ovarian cancer plasma samples [30,31]. More controversial findings are from studies reporting no differences in the plasma content of LPA between ovarian cancer and control patients [32,33].

Controlled experiments have shown that the content of LPA is artificially increased during sample processing and analysis, e.g., the use of acids for extraction [12], incubating blood samples at room temperature [13], and mass analysis without prior separation of LPA from other lysophospholipids [22]. However, this well-known concept has not been acknowledged in ovarian cancer research wherein many studies have used either acid for the extraction process [8,23,24,25,26,27], or mass analysis without prior separation of LPA [28]. This interference may not directly cause the increase of LPA in cancer patients, but should contribute to the high variability of LPA content measured in control patients, ranging from 0.06 to 4.7 µmol/L. Moreover, the artificial conversion may be increased in patients with a higher initial content of these lysophospholipids.

The confounding factors reported from controlled experiments do not fully cover all possible variations that may arise in analyzing clinical samples. For example, it is often assumed that LPA content does not change in plasma when stored at −80 °C. However, the stability of LPA in samples stored for several years has not been assessed, which has been the case in most ovarian cancer studies. The role of EDTA in influencing LPA content is also not clear, in that some studies reported that treatment with EDTA stopped the accumulation of LPA during incubation at room temperature [33,34], whereas the results of other studies contradicted these findings [11,13]. Since the concentration of EDTA varies depending on the blood volume collected in EDTA-coated tubes, the role of EDTA has to be better clarified. Overall, prior studies have not ensured biomarker stability, which significantly weakens the claimed potential of LPA as a biomarker for ovarian cancer. Therefore, propagation of this unverified notion by other publications should be reduced.

In the current study, we show that (1) the content of LPA increased during long term storage in the freezer, (2) the artificial increase in LPA at room temperature was affected by the amount of EDTA, and (3) this increase was most prominent for LPA when compared to other lysophospholipids. We believe that these factors, resulting in the artificial increase of LPA, may potentially explain the contradictory increase of LPA observed in ovarian cancer.

Clinical studies are subjected to regulations on enlisting human patients and collecting and processing human samples, which often differ depending on study groups or controls. Most prior publications on the diagnostic role of LPA in ovarian cancer provided details about the recruitment process of cancer patients and reported that a minimum of two years was spent collecting specimens from these patients. Plasma separated from the collected samples was stored in freezers until the collection procedure was complete, providing a minimum two year storage period for LPA concentrations to increase. However, it is neither clearly stated whether the control samples were also collected over the same time interval, nor if the control samples were matched against cancer samples with similar collection times. From a practical stand point, enlisting non-cancer patients is more challenging than cancer patients, the target study group. As such, healthy volunteers are either directly recruited by investigators, or their “convenient” samples that are not specifically collected for the study, are often used as the control group; these samples undergo different processing procedures than cancer samples and/or have limited information on the sample collection and processing procedures.

If the control samples were collected over a similar period of time as the collection of cancer samples, the control samples should show a substantial time-dependent distribution, which can be seen in Figure 2. Interestingly, there is a typical pattern seen in studies showing a substantial increase in LPA levels in ovarian cancer: control samples with a narrower distribution range and cancer samples with a wider distribution range [8,14,24,25,30,31]. On the contrary, literature that demonstrated no differences in LPA levels between the cancer and control samples showed a similar distribution range between the two groups [33], which is consistent with our argument that storage time, rather than cancer pathology, may be responsible for the increased amount of LPA detected in cancer samples.

The blood samples in different groups may also have different incubation times, which heavily depends on the delivery time of the drawn blood samples from the phlebotomists to the investigators. Again, if the control samples are collected directly by investigators, controls will have significantly shorter incubation times, and thus will have a lower LPA content. In our study, we did not have direct control of the sample collection from healthy controls, whereas we had a more regulated control on obtaining samples from the cancer and benign patients. The acquisition time of control samples may have been longer than for benign and cancer samples, potentially explaining our data wherein the control LPA levels were higher than in the benign and cancer LPA levels.

Another confounder that is very difficult to control is the EDTA concentration in collection tubes. Common blood collecting tubes contain enough EDTA to give approximately 5 mM EDTA when the blood is fully collected. Therefore, the concentration of EDTA will be greater than or equal to 5 mM depending on the volume of blood added. We have shown that the increase in LPA content in blood incubated with 5 mM EDTA was greater than in blood incubated with 10 mM EDTA. Thus, variations in blood volume during sample collection can lead to differences in the time-dependent increase of LPA during incubation.

Very few studies have looked into the effects of EDTA on the formation of LPA, or lack thereof. Nakamura et al. described the production of LPA as inevitable, due to a variety of different pathways that involve platelet-mediated and/or enzyme-mediated mechanisms [35]. Yatomi et al. showed that ATX was the main cause of the increase in plasma LPA [36]. In order to confirm the role of ATX in LPA formation, we added ATX inhibitors to whole blood and plasma in the presence of 5 mM EDTA, which resulted in the lack of LPA increase, confirming the significance of ATX in reducing the artificial increase of LPA. This finding further explains the influence of an increasing EDTA concentration on the inhibition of LPA generation that we observed. The Zn^2+^ chelation effect of EDTA, which is an essential cofactor of ATX function, may decrease the production of LPA via ATX [37]. Therefore, addition of ATX inhibitors may be a potential solution to minimize artificial LPA increase in collected blood.

Since most publications do not provide detailed information regarding the procedure for sample collection and preparation, our discussion is limited to postulation. Therefore, we cannot confirm whether or not LPA is indeed increased in ovarian cancer. However, our data clearly shows that there are major confounding factors that previous studies have ignored, yet could have interfered with their LPA analysis. There may be other factors that affect short term and/or long term stability of LPA. It may be possible that the increased LPA found in cancer patients is not due to the cancer pathology, but rather due to the differences in the procedure of blood sample collection and processing, depending on subject groups. This instability makes the LPA analysis highly challenging for use in clinical research, where rigorous experimental controls are compromised by the limits and regulations of handling human samples.

## 4. Materials and Methods

### 4.1. Reagents

Reagent-grade chemicals and HPLC-grade solvents were purchased from major commercial suppliers (Fisher Scientific, Hampton, NH, USA, and Sigma Aldrich, St. Louis, MO, USA). Internal standards, LPA(17:0), LPC(17:1), LPE(17:1), and LPI(17:1) were purchased from Avanti Polar Lipids (Alabaster, AL, USA). Milli-Q water (Burlington, MA, USA) was used throughout all experiments. ATX inhibitor, 4-[3-Oxo-3-(2-oxo-2,3-dihydrobenzoxazol-6-yl)propyl]piperazine-1-carboxylic acid 3,5-dichlorobenzyl ester (PF-8380) and [4-(Tetradecanoylamino)benzyl]phosphonic acid (S32826) were purchased from Cayman Chemical (Ann, Arbor, MI, USA).

### 4.2. Plasma Samples

All patient-derived specimens were collected under protocols approved by the Institutional Review Board at the Feinstein Institute for Medical Research (The IRB protocol number is 10-193A). Informed consent was not obtained as no individual data was collected. This study used stored plasma samples, which were collected from three groups: patients with confirmed ovarian cancer (cancer, *n* = 20, 16 from stage III and IV, and 4 from stage I and II), benign ovarian tumors (benign, *n* = 23), and healthy non-cancer (control, *n* = 18). Plasma was separated from the blood specimen, which was collected in EDTA-containing tubes after centrifugation and stored at −80 °C until later processing. All obtained blood samples were processed within 4 h of blood drawing. The average plasma sample storage time was similar between the three groups. In order to test the effects of blood incubation time, the concentration of EDTA, and the addition of ATX inhibitors (PF-8380 and S32826) on lysophospholipids, freshly collected blood samples from healthy volunteers were used.

### 4.3. Blood Incubation Analysis

Blood drawn from healthy volunteers was treated with either 5 mM or 10 mM EDTA and was incubated at room temperature for up to 4 h. After the incubation period, plasma was separated using centrifugation. We also analyzed a second group of control blood plasma, which was separated from EDTA-treated whole blood immediately after blood draw. This allowed us to assess the effect of EDTA on lysophospholipids. In addition, blood samples containing 5 mM EDTA were treated with 1 µM of an ATX inhibitor, either PF-8380, or S32826, and were incubated at room temperature for 4 h. At the end of the incubation period, the plasma samples were stored at −80 °C overnight and extracted as described below.

### 4.4. Extraction of Phospholipids

Lipids were extracted from the plasma samples following the published method [12]. Briefly, 100 μL of plasma was extracted with 1.5 mL of methanol in the presence of 1.7 nmol of LPC(17:1), 0.3 nmol of LPE(17:1), 0.1 nmol of LPI(17:1), and 0.45 nmol of LPA(17:0) as internal standards. The mixture was vortexed for 2 minutes, incubated for 10 min at 4 °C, and centrifuged for 10 minutes at 16,000× *g*. The supernatant was decanted and evaporated to dryness using N_2_. The residue was reconstituted in a 0.1 mL of solution containing isopropanol (IPA), t-butyl methyl ether (MTBE), and aqueous ammonium formate (94 mM) in a 34:17:5 (*v*:*v*:*v*) ratio, respectively. Finally, 20 μL of the solution was injected into the HPLC-MS for analysis. The overall recovery rate of LPA(17:0) through the procedure was 88%.

### 4.5. HPLC-MS Analysis

The phospholipid mixture was analyzed using normal-phase HPLC-MS [38,39]. Eluent A was created using IPA, MTBE, and aqueous ammonium formate in a 340:170:50 (*v*:*v*:*v*) ratio (94 mM, pH ~2.5) with eluent B containing methanol. The gradients used for the 35 min chromatogram were as follows: 100% A for 18 min, 100% A to 20% A over 6 min, 20% A for 3 min, 20% A to 100% A over 1 min, and hold 100% A for 7 min. The flow rate was 0.3 mL/min and the column temperature was 30 °C.

MS and MS/MS data were obtained with an LTQ XL spectrometer (Thermo Scientific, San Jose, CA, USA) operated in the negative ion mode [21]. Data was then processed using Thermo Xcalibur software (version 2.2, Waltham, MA, USA) [21,40]. Retention time, MS, and MS/MS data were compared to the control to identify the individual species. The concentrations of the lysophospholipid species were calculated by comparing their peak areas to the areas of corresponding standard lysophospholipids.

### 4.6. Statistical Analysis

Data were expressed as the mean ± standard deviation for continuous variables. The concentrations of lysophospholipid species were compared using the Mann–Whitney U test for continuous variables, as appropriate. To assess the effect of storage time on the lysophospholipid levels, a regression analysis was performed using linear regression and Spearman’s correlation coefficients. *p* values less than 0.05 were considered to be statistically significant. All analyses were performed using the SPSS software package (version 25.0 J SPSS, IBM, Chicago, IL, USA).

## 5. Conclusions

LPA is a dynamic molecule whose content readily changes in response to changes in its environment. This sensitive response makes this molecule attractive as an early marker of various pathological conditions, but also increases the chance for false positive results due to artificial increases during sample collection, storage, and analysis. We show that plasma LPA content increased over time during the incubation of blood at room temperature and during the long-term storage of frozen plasma samples. Additionally, the amount of EDTA also interfered with these changes. These interfering factors are difficult to accommodate for when handling clinical samples, which are collected and handled using non-researcher-controlled procedures. However, previous studies have not considered these factors, creating inconsistent reports on the relationship between LPA and ovarian cancer. As such, the diagnostic role of LPA in ovarian cancer needs to be reconsidered in any future research with carefully designed settings that appropriately account for these confounding factors.

## Figures and Tables

**Table 1:** Lysophospholipids (LPPL) (μmol/L) in control, patients with benign ovarian tumor, and patients with ovarian cancer groups (*n* = 18 for control, *n* = 23 for benign, *n* = 20 for cancer).

LPPL Species	Control (mean ± SD)	Benign (mean ± SD)	Cancer (mean ± SD)	*p* Value (Control vs. Benign)	*p* Value (Control vs. Cancer)	*p* Value (Benign vs. Cancer)
LPA(18:2)	0.26 ± 0.20	0.12 ± 0.07	0.10 ± 0.09	0.004	0.001	0.197
LPA(16:0)	0.09 ± 0.07	_	_	_	_	_
LPI(18:0)	10.4 ± 6.63	4.79 ± 5.25	3.19 ± 2.13	0.004	0.001	0.992
LPI(20:4)	2.28 ± 1.09	3.38 ± 1.97	3.69 ± 2.38	0.046	0.038	0.846
LPC(16:0)	178.8 ± 47.6	143.4 ± 45.2	121.4 ± 44.2	0.036	0.001	0.108
LPC(18:0)	83.0 ± 31.2	60.5 ± 23.4	51.0 ± 19.0	0.026	0.001	0.158
LPC(18:2)	64.4 ± 19.6	70.2 ± 39.2	66.7 ± 37.1	0.937	0.682	0.808
LPC(20:4)	17.0 ± 7.36	27.6 ± 18.1	30.5 ± 19.1	0.052	0.007	0.527
LPC(22:6)	4.11 ± 2.38	6.78 ± 4.50	9.20 ± 6.36	0.049	0.001	0.181
LPE(16:0)	1.74 ± 0.64	1.55 ± 0.62	1.57 ± 0.76	0.344	0.320	0.981
LPE(18:0)	2.70 ± 1.20	2.06 ± 0.95	2.11 ± 0.96	0.043	0.054	0.770
LPE(18:2)	3.08 ± 1.30	2.45 ± 0.96	2.12 ± 1.13	0.078	0.014	0.233
LPE(20:4)	1.48 ± 0.70	1.73 ± 0.49	1.78 ± 0.70	0.055	0.121	0.981
LPE(22:6)	0.99 ± 0.45	1.51 ± 0.42	1.86 ± 0.97	<0.001	0.001	0.436

## Supplementary Materials

The following are available online at https://www.mdpi.com/2072-6694/11/4/520/s1, Figure S1: Regression analysis of other individual lysophospholipids species as a function of the duration of storage time in the control samples, Figure S2: Whole blood and plasma incubation for 4 h at room temperature in LPA(18:2), LPA(16:0) and LPA(20:4), Table S1: Summary of previous studies on LPA in ovarian cancer.
