# Supplementary material for: Challenges and Inconsistencies in Using Lysophosphatidic Acid as a Biomarker for Ovarian Cancer
Source: Cancers (Basel). 2019 Apr 11;11(4):520. doi: 10.3390/cancers11040520 (PMC6521627; doi:10.3390/cancers11040520)
Supplement: Supplementary file 1 [file cancers-11-00520-s001.pdf]

Supplementary Materials

Challenges and Inconsistencies in Using Lysophosphatidic Acid as a Biomarker for Ovarian Cancer

Tsukasa Yagi, Muhammad Shoaib, Cyrus E. Kuschner, Mitsuaki Nishikimi, Lance B. Becker, Annette T. Lee and Junhwan Kim

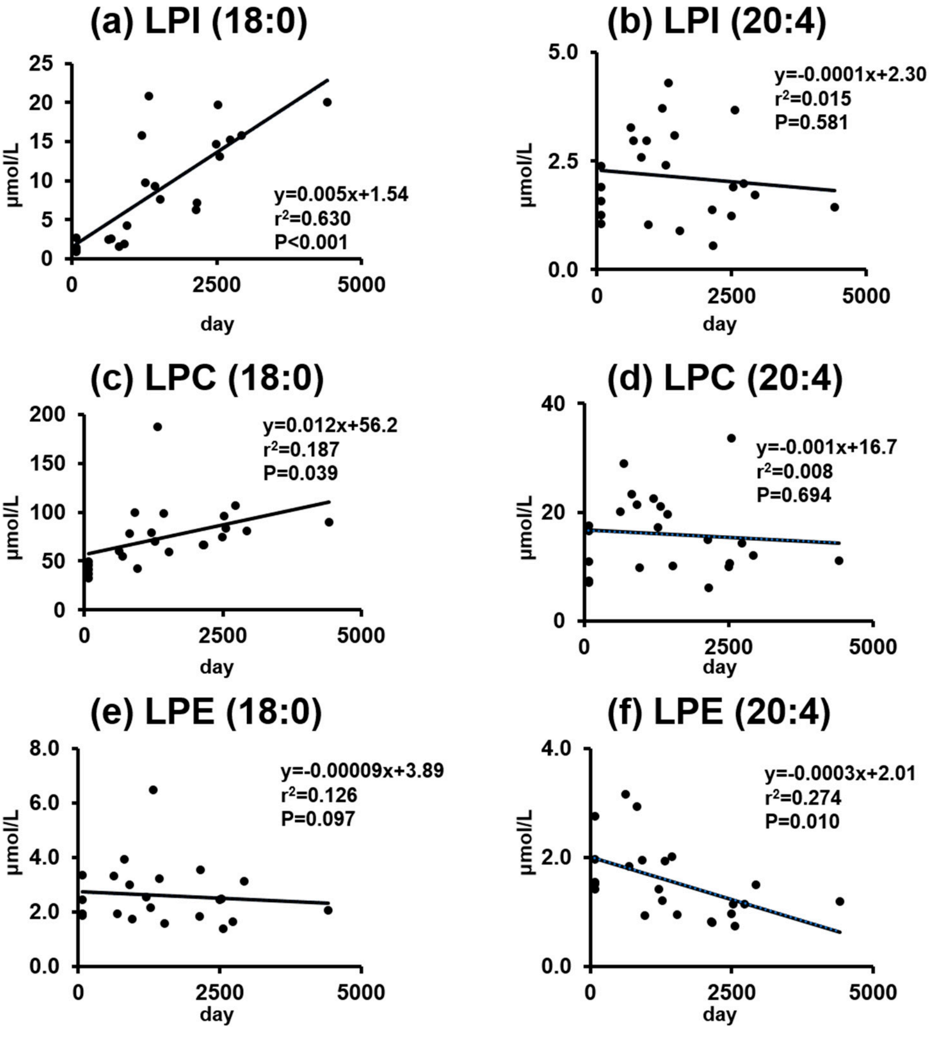

Figure S1. Regression analysis of other individual lysophospholipids species as a function of the duration of storage time in the control samples. The contents of LPI(18:0) and LPC(18:0) are significantly increased as a function of storage times (**a**,**c**), whereas the content of LPE(20:4) is significantly decreased (**f**). The contents of LPI(20:4), LPC(20:4), and LPE(18:0) are not significantly changed (**b**,**d**,**e**).

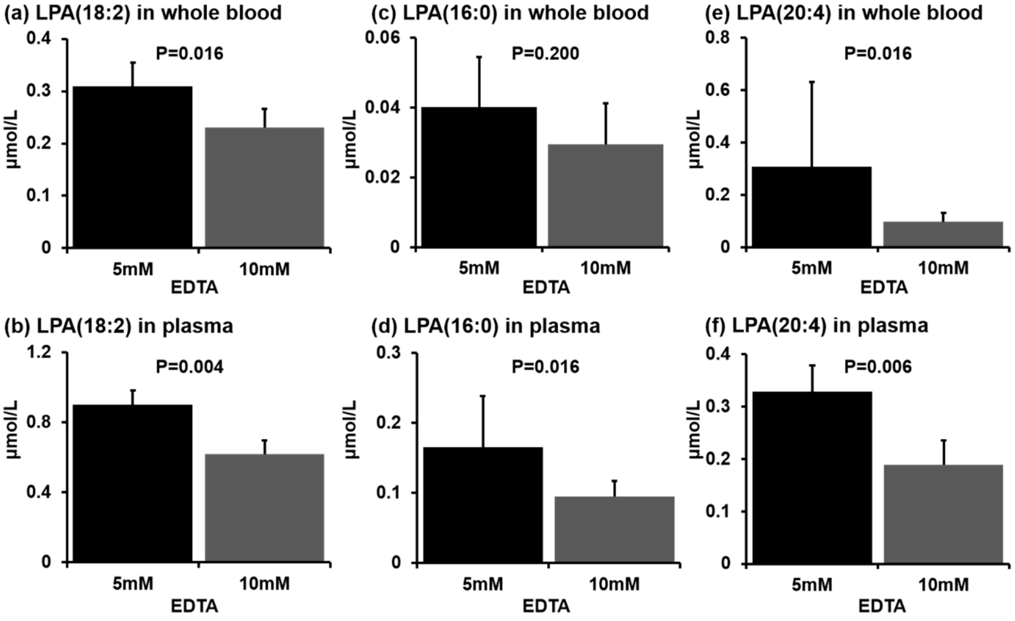

Figure S2. Whole blood and plasma incubation for 4 h at room temperature in lysophosphatidic acid LPA(18:2), LPA(16:0) and LPA(20:4). The increases in the contents of LPA(18:2), LPA(16:0), and LPA(20:4) in whole blood (**a**,**c**,**e**) and in plasma (**b**,**d**,**f**) are greater in the presence of 5 mM ethylenediaminetetraacetic acid (EDTA) compared to 10 mM EDTA.

Table S1. Summary of previous studies on LPA in ovarian cancer.

Reference	Country	Sample Collection		Method		LPA (μmol/L)			Sample Number		
		CAN	CON	acid	analysis	CON	BEN	CAN	CON	BEN	CAN
[1]	USA	2–3 years	same	+	GC-MS	0.6	2	8.6	48	17	48
[2]	USA	2.2 years	same	+	MS	1.1	-	3.3	10	-	9
[3]	USA	NM [1]	NM	-	LC-MS	0.7	-	0.8	32	-	49
[4]	Korea	NM	NM	+	MS	4.7	-	8.1	4	-	3
[5]	USA	2–3 years	same	+	MS	0.9	-	2.7	27	-	45
[6]	Slovenia	NM	NM	-	LC-MS	3.0	8.0	8.4	78	142 [2]	
[7]	Slovenia	NM	NM	-	LC-MS	2.9	8	8.4	55	65	50
[8]	USA	NM	NM	-	LC-MS	2.4	2.6	2.4	25	27	26
[9]	Czech	3 years	same	+	CE analysis	2.9	7.7	17.0	40	30	60
[10]	China	3.3 year	same	-	phosphorous	2.4	2.6	6.4	36	36	36
[11]	Turkey	NM	NM	+	GC-MS	0.6	1.6	4.3	50	74	87
[12]	Czech	5 years	same	+	CE analysis	1.9	6.2	11.5	27	51	81
[13]	China	3.5 years	NM	-	ELISA	0.6	1.2	3.9	30	40	80
[14]	China	~ 2 years		-	ELISA	-	1.8	5.3	-	100	100
[15]	China	1 year	same	-	ELISA	1.9	1.9	5.3	75	70	98

1, Not mentioned; 2, breakdown was not mentioned; CAN, cancer; CON, control; BEN, benign.

Reference

1. Xu, Y.; Shen, Z.; Wiper, D.W.; Wu, M.; Morton, R.E.; Elson, P.; Kennedy, A.W.; Belinson, J.; Markman, M.; Casey, G. Lysophosphatidic acid as a potential biomarker for ovarian and other gynecologic cancers. *JAMA* **1998**, *280*, 719–723.
2. Xiao, Y.; Chen, Y.; Kennedy, A.W.; Belinson, J.; Xu, Y. Evaluation of plasma lysophospholipids for diagnostic significance using electrospray ionization mass spectrometry (esi-ms) analyses. *Ann. N. Y. Acad. Sci.* **2000**, *905*, 242–259.
3. Baker, D.L.; Morrison, P.; Miller, B.; Riely, C.A.; Tolley, B.; Westermann, A.M.; Bonfrer, J.M.; Bais, E.; Moolenaar, W.H.; Tigyi, G. Plasma lysophosphatidic acid concentration and ovarian cancer. *JAMA* **2002**, *287*, 3081–3082.

4. Yoon, H.-R.; Kim, H.; Cho, S.-H. Quantitative analysis of acyl-lysophosphatidic acid in plasma using negative ionization tandem mass spectrometry. *J. Chromatogr. B* **2003**, *788*, 85–92.
5. Sutphen, R.; Xu, Y.; Wilbanks, G.D.; Fiorica, J.; Grendys, E.C., Jr.; LaPolla, J.P.; Arango, H.; Hoffman, M.S.; Martino, M.; Wakeley, K.; et al. Lysophospholipids are potential biomarkers of ovarian cancer. *Cancer Epidemiol. Biomark. Prev. Publ. Am. Assoc. Cancer Res. Cosponsored Am. Soc. Prev. Oncol.* **2004**, *13*, 1185–1191.
6. Pozlep, B.; Meleh, M.; Kobal, B.; Verdenik, I.; Osredkar, J.; Kralj, L.Z.; Meden-Vrtovec, H. Use of lysophosphatidic acid in the management of benign and malignant ovarian tumors. *Eur. J. Gynaecol. Oncol.* **2007**, *28*, 394–399.
7. Meleh, M.; Pozlep, B.; Mlakar, A.; Meden-Vrtovec, H.; Zupancic-Kralj, L. Determination of serum lysophosphatidic acid as a potential biomarker for ovarian cancer. *J. Chromatogr. B Anal. Technol. Biomed. Life Sci.* **2007**, *858*, 287–291.
8. Murph, M.; Tanaka, T.; Pang, J.; Felix, E.; Liu, S.; Trost, R.; Godwin, A.K.; Newman, R.; Mills, G. Liquid chromatography mass spectrometry for quantifying plasma lysophospholipids: Potential biomarkers for cancer diagnosis. *Methods Enzymol.* **2007**, *433*, 1–25.
9. Sedlakova, I.; Vavrova, J.; Tosner, J.; Hanousek, L. Lysophosphatidic acid: An ovarian cancer marker. *Eur. J. Gynaecol. Oncol.* **2008**, *29*, 511–514.
10. Cao, X.Y. The applicable value of conbined detection of lpa, ca125 and afp in the early diagnosis of ovarian cancer. *Lab. Med. Clin.* **2008**, *5*, 1430–1431.
11. Bese, T.; Barbaros, M.; Baykara, E.; Guralp, O.; Cengiz, S.; Demirkiran, F.; Sanioglu, C.; Arvas, M. Comparison of total plasma lysophosphatidic acid and serum ca-125 as a tumor marker in the diagnosis and follow-up of patients with epithelial ovarian cancer. *J. Gynecol. Oncol.* **2010**, *21*, 248–254.
12. Sedlakova, I.; Vavrova, J.; Tosner, J.; Hanousek, L. Lysophosphatidic acid (lpa)-a perspective marker in ovarian cancer. *Tumour Biol.* **2011**, *32*, 311–316.
13. Wang, D.I.; Chen, L.X.; Liao, X.I.; Li, X.J.; Wu, Q.H. The relationship of lysophosphatidic acid and mmp-2 in diagnosing epithelial ovarian carcinoma. *Proc. Clin. Med.* **2013**, *22*, 403–405.
14. Zhang YJ, Cao LY, Fu ZZ, Wang YJ, Wang GX, Gu T. Clinical significance of plasma lysophosphatidic acid levels in the differential diagnosis of ovarian cancer. *J. Cancer Res. Ther*. **2015**, *11*, 375–380.
15. Cao, L.; Zhang, Y.; Fu, Z.; Dong, L.; Yang, S.; Meng, W.; Li, Y.; Zhang, W.; Zhang, J.; Zheng, C.; et al. Diagnostic value of plasma lysophosphatidic acid levels in ovarian cancer patients: A case-control study and updated meta-analysis. *J. Obstet. Gynaecol. Res.* **2015**, *41*, 1951–1958.

© 2019 by the authors. Licensee MDPI, Basel, Switzerland. This article is an open access article distributed under the terms and conditions of the Creative Commons Attribution (CC BY) license (http://creativecommons.org/licenses/by/4.0/).
